# Online psychosocial group intervention for adolescents with a chronic illness: A randomized controlled trial

**DOI:** 10.1016/j.invent.2021.100447

**Published:** 2021-08-20

**Authors:** Miriam Douma, Heleen Maurice-Stam, Bianca Gorter, Bregje A. Houtzager, Hestien J.I. Vreugdenhil, Maaike Waaldijk, Lianne Wiltink, Martha A. Grootenhuis, Linde Scholten

**Affiliations:** a Amsterdam UMC, University of Amsterdam, Emma Children's Hospital, Psychosocial Department, Meibergdreef 9, 1105 AZ Amsterdam, the Netherlands; b Princess Máxima Center for Pediatric Oncology, Psychosocial Department, Heidelberglaan 25, 3584 CS Utrecht, the Netherlands; c DeKinderKliniek, Medical Psychology, Hospitaaldreef 29, 1315 RC Almere, the Netherlands; d Deventer Hospital, Medical Psychology, Nico Bolkesteinlaan 75, 7416 SE Deventer, the Netherlands; e Amsterdam UMC, University of Amsterdam, location VUmc, Medical Psychology De Boelelaan 1117/1118, 1081 HV Amsterdam, the Netherlands; f St. Jansdal Hospital, Medical Psychology, Wethouder Jansenlaan 90, 3844 DG Harderwijk, the Netherlands; g Canisius Wilhelmina Hospital, Medical Psychology, Weg Door Jonkerbos 100, 6532 SZ Nijmegen, the Netherlands

**Keywords:** Psychiatry/psychology, Psychosocial issues, Developmental/behavioral issues

## Abstract

**Objective:**

The present study aimed to evaluate the efficacy of *Op Koers Online*, a protocolled online psychosocial group intervention for adolescents with a chronic illness (CI).

**Methods:**

Adolescents (12–18 years) with different types of CI (*N* = 59; Mean age = 15.1 years, SD = 1.7; 54% female) participated in a parallel multicenter randomized controlled trial comparing *Op Koers Online* (*N* = 35) with a waitlist control group (*N* = 24). Assessments (online questionnaires) took place at baseline (T0), 6-months (T1) and 12-months follow-up (T2). Primary outcomes were internalizing and externalizing behavioral problems and disease-related coping skills. Health-Related Quality of Life was secondary. Efficacy was tested with linear mixed models.

**Results:**

Compared to the control group, the intervention had a significant positive effect (*p* < .05) on disease-related coping skills T1 vs T0 (use of relaxation, β = 0.68; social competence, β = 0.57) and T2 vs T0 (information seeking, β = 0.61), and on HRQoL (social-, school-, psychosocial functioning and total HRQoL) T1 vs T0 (β = 0.52 to β = 0.60). No intervention effects on internalizing and externalizing behavioral problems were found.

**Conclusion:**

The results of this randomized controlled trial indicate a positive effect of *Op Koers Online*. The intervention had beneficial effects on disease-related coping skills and HRQoL.

**Practice implications:**

The next step is to implement *Op Koers Online* for adolescents in clinical practice.

## Highlights

• Adolescents with a chronic illness are at risk for psychosocial problems.
• Efficacy of a psychosocial group intervention *Op Koers Online* was studied in a RCT.
• The intervention had beneficial effects on quality of life.
• The intervention had beneficial effects on the use of adaptive coping skills.
• The next step is to implement the intervention *Op Koers Online* in clinical practice.

## 1 Introduction

Children and adolescents growing up with a chronic illness (CI; e.g. asthma, diabetes) are at risk for emotional- and social problems such as feeling down and isolating oneself (Sawyer et al., 2007; Pinquart, 2013; Pinquart and Teubert, 2012). When untreated, these problems can cause mental disorders such as depression (Suris et al., 2008). During adolescence, patients have the challenge of becoming autonomous, which is extra demanding for those with a CI (Suris et al., 2004). Considering this, interventions that support adolescents with a CI and teach them how to cope with their illness are essential.

Cognitive Behavioral Therapy (CBT) is known as an effective evidence-based psychological treatment for youth with a CI (Compton et al., 2004; Johansson et al., 2019) in improving psychosocial wellbeing (Serlachius et al., 2016) and in teaching positive coping skills (Dorris et al., 2017). Coping skills are important mediators of effects of a CI on adaptive psychosocial functioning (Plante et al., 2001).

Offering CBT interventions via the internet is upcoming because of the logistical and practical benefits (Dever Fitzgerald et al., 2010; Ritterband and Thorndike, 2012). For example, patients do not need to visit the hospital because they can participate from home, which is also a great advantage for countries with sparse populations. Moreover, an online environment is appealing to adolescents specifically, since they are generally used to being online (van Beugen et al., 2014; Ahola Kohut et al., 2014). Results of online CBT interventions on improving psychological functioning and decreasing disease-related impact are promising. A meta-analysis of the results of randomized controlled trials (RCT) about the effect of online CBT interventions compared to control conditions (not therapeutic treatment or treatment as usual) showed reduction of depressive symptoms, anxious symptoms and general distress. Between-group effect sizes were 0.17 for anxiety and 0.21 for depression and general distress (van Beugen et al., 2014).

Online CBT interventions can be offered in individual or group format. Group interventions have important advantages, such as the possibility to share emotions and experiences with others in a similar situation and the fact that therapists can treat more patients simultaneously (Plante et al., 2001; Heath et al., 2018). Moreover, group interventions are proven to be effective in teaching coping skills and improving knowledge about symptom reduction and disease-related problem-solving (Plante et al., 2001; Clarke and DeBar, 2010). However, studies that evaluate online group therapies for adolescents with a CI are scarce (Bekker et al., 2017).

Most existing (CBT) group interventions for adolescents with a CI, face to face as well as online interventions, are focused on a specific illness (Palermo et al., 2016; Stinson et al., 2016), such as epilepsy (Dorris et al., 2017). However, most of the psychosocial problems (e.g. anxiety and depressive symptoms) are the same across illnesses. Generic consequences of having a CI (e.g. feeling different, dealing with food or social restrictions, taking medication) cause psychosocial problems (Plante et al., 2001). A generic approach that focuses on psychosocial problems associated with the CI rather than the CI itself is therefore suitable and would allow for patients with rare illnesses to participate in a group intervention. Besides, it offers therapists the possibility to treat more patients simultaneously which can be cost-effective.

The intervention in the current study, *Op Koers Online* (English: *On Track Online*) is an online CBT group intervention (chat, without use of video) for adolescents with different types of CI based on the already existing face-to-face interventions *Op Koers* for children, adolescents and parents (Last et al., 2007; Scholten et al., 2013). *Op Koers* face-to-face showed positive results on improving psychosocial functioning (parent-reported internalizing problems and child-reported externalizing problems) and on use of the coping skills information seeking, social competence and positive thinking (Scholten et al., 2013). A pilot study of Op Koers Online for adolescent survivors of childhood cancer showed promising results on feasibility (Maurice-Stam et al., 2014). Satisfaction rates of both course leaders and participants were high Another pilot study of Op Koers Online for adolescents with a CI showed promising results on efficacy: participant's use of several coping skills and aspects of Health-Related Quality of Life (HRQoL) improved after following the intervention (Douma et al., 2019). The pilot studies did not include a control group, therefore more research was needed to establish the effects of the intervention.

In a RCT we aimed to answer the following research question: Is *Op Koers Online* for adolescents with a CI an efficacious intervention? We hypothesized that *Op Koers Online* for adolescents had a positive effect on adolescent's internalizing and externalizing behavioral problems, disease-related coping skills and HRQoL.

## 2 Methods

### 2.1 Study design

A multicenter parallel RCT comparing the intervention to a control group (waitlist control group) was designed in accordance with the Standard Protocol Items: Recommendations for Intervention Trials Checklist (CONSORT-Checklist). In this RCT we used the assessments (online questionnaires) that were completed at baseline (before randomization; T0), at 6-months (T1) and 12-months (T2) follow-up from baseline. Full details of the study protocol and the intervention were reported previously by Douma et al. (2018) (registry number ISRCTN83623452). Approval of the Medical Ethical Committee of the Amsterdam University Medical Centers was obtained for this study. Participants from both the control and intervention group received care-as-usual and were not prevented to seek individual psychosocial treatment.

#### 2.1.1 Procedure

The study was conducted between July 2016 and April 2019. Participants were recruited between September 2016 and June 2018 from outpatient clinics of nine participating hospitals via information letters and pamphlets. Healthcare professionals were asked to invite adolescents in person to participate in the study. Additional nationwide recruitment was done via patient associations and online advertisement. Interested adolescents could use a reply form or send an e-mail. After a positive reply, adolescents (and their parents) were phoned to assess eligibility and to obtain informed consent. Inclusion criteria were aged between 12 and 18 years with a physical CI diagnosis, according to the following criteria set forth by Mokkink et al.: 1) onset between ages 0 and 18; 2) diagnosis based on medical scientific knowledge; 3) the illness is not (yet) curable; and 4) the illness has been present for at least three months or at least three episodes have occurred in the last year (Mokkink et al., 2008). Furthermore, having access to a laptop/computer/tablet with internet connection was necessary to participate in the intervention and to complete questionnaires. Exclusion criteria were having cognitive disabilities or language problems which limited the ability to participate in the intervention and/or to fill out questionnaires.

#### 2.1.2 Randomization

The randomization into intervention and control group was carried out by an independent IT worker from a company for e-health development who administers the website for *Op Koers Online*. Block randomization with block size four was performed, based on a previously generated randomization schedule with allocation ratio 1:1. Because the recruitment period was spread out over time, randomization was done at five time points. In case of an incomplete randomization block, participants were assigned to the intervention group, to assure enough participants to give the group intervention. When randomized in the intervention group, the researcher called the participant to schedule the intervention. Participants in the control group were given the opportunity to participate in the intervention after the final follow-up assessment. The researchers were not blinded to group assignment.

#### 2.1.3 Intervention

*Op Koers Online* consists of eight weekly 90-minutes sessions and a booster session 4 months after the last session. The goal of the intervention is to prevent and/or reduce psychosocial problems by teaching the use of engaged coping skills using CBT techniques, such as cognitive restructuring and relaxation. Five coping skills were taught; 1) information seeking (how to find information about the illness) and providing information about the illness (how to communicate about the illness to peers), 2) use of relaxation techniques in stressful situations, 3) increasing knowledge of self-management and medical compliance, 4) improving social competence and 5) positive thinking (cognitive restructuring) (Last et al., 2007; Douma et al., 2018). The learning goals of the intervention and examples of learning activities are shown in Table 1.

**Table 1:** Learning goals and examples of learning activities of Op Koers Online for adolescents.

		Learning goals				
		Information seeking and giving about the illness	Use of relaxation during stressful situations	Increase knowledge of self-management and compliance	Enhancement of social competence	Positive thinking (cognitive restructuring)
Examples of learning activities of Op Koers Online for adolescents	Instruction/modeling Session^a^	Education about sources of information Session 3	Relaxation exercise (MP3) Session 2	Group discussion about own treatment and (non-)compliance Session 4	Video and group discussion: how and what do you tell others about your illness Session 5 and 6	Thinking-Feeling-Doing game Session 1, 7 and 8
	Reinforcement/practice (homework)	Write down questions you have, and look for answers	Practice the relaxation exercise	Write down situations for non-compliance and how to improve compliance	Think of what CAN you do (instead of CANNOT) and write down your story for the other group members	Write down positive adjustments for negative thoughts

a The session that focused on the specific learning goal was mentioned here. However learning goals are applied throughout the whole course (for example: use of relaxation techniques and Thinking-Feeling-Doing are discussed in multiple sessions).

**Fig. 1:**
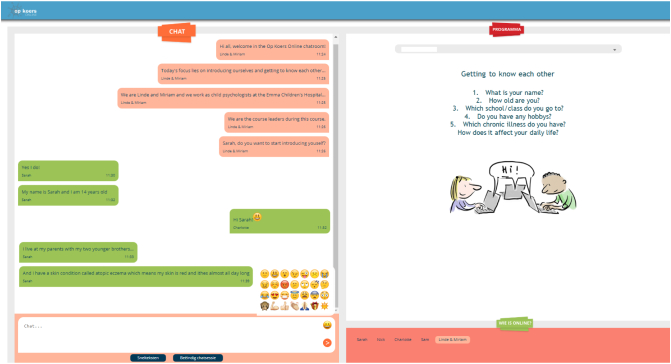
Chat room of Op Koers Online. Left screen: chat text; right screen: information screens/videos/exercises; strip below (left): field where participant writes their text with possible use of the emojis; strip below (right): names of participants who are present in the chatroom*. Note: These participants and text in this chat room are fictitious.*

Sessions took place at a scheduled time in a secured chatroom (Fig. 1) with three to six participants and two qualified psychologists (course leaders) who carry out the protocolled intervention. The intervention was designed without the use of a video camera to ensure anonymity as much as possible and keep the threshold for participation low. Turn taking during the sessions was managed by the course leaders. After each session, course leaders filled out a log providing information about the session: particularities of participant's absence or situation, any technological issues and whether or not course leaders followed the intervention protocol. The log was checked by the coordinating researcher. Assessment of the log did not show any major deviations of the intervention protocol.

All course leaders (*N* = 22 in total; all female and psychologist) from nine participating hospitals are extensively trained during an 8-hour workshop in using the detailed intervention protocol. The workshop was led by the coordinating researchers of the study and took place in the Emma Children's Hospital. It included a theoretical part and exercises to learn how to use the website.

Participants log on to the website (www.opkoersonline.nl) to enter the chatroom and their own personal environment to complete homework assignments. To improve adherence, participants received a small gift (e.g. memory game) after the last session as a reward for participating and completing homework assignments.

### 2.2 Measures

#### 2.2.1 Primary outcomes

Behavioral problems were assessed with the Child Behavior Checklist (CBCL; 6–18 years, parent report) and the Youth Self Report (YSR; 11–18 years) (Verhulst et al., 1996; Verhulst et al., 1997), consisting of items about problem behavior during the past six months. Two broadband scales were used in the present study: internalizing problems and externalizing problems. Internalizing problems (range 0–42) included the subscales anxious-depressed and withdrawn-depressed. Items from the subscale somatic complaints were disregarded in the analyses (Perrin et al., 1991). Externalizing problems (CBCL range 0–70, YSR range 0–64) included the subscales rule-breaking behavior and aggressive behavior. Higher scores indicate more problem behavior. The internal consistency of the internalizing and externalizing scales was satisfactory across time points, with Cronbach's α = 0.78 to α = 0.99.

Disease-related coping skills were assessed with the Op Koers Questionnaire (OKQ) (Last et al., 2007; Douma et al., 2019). Adolescents were asked to what extent (1 “always/almost always” to 4 “almost never/never”) they agree with statements about the use of coping skills taught in *Op Koers Online* (e.g. “I know how to get answers about my disease”). The items are divided into five subscales: information seeking (6 items), relaxation (3 items), social competence (6 items), positive thinking (3 items) and medical compliance (8 items). Higher scores reflect more use of engaged coping skills. Internal consistency was moderate to satisfactory across time points, with Cronbach's α = 0.61 to α = 0.92, except for relaxation at T0 (α = 0.51) and medical compliance at T0 (α = 0.45). The last was excluded from the analyses.

#### 2.2.2 Secondary outcomes

HRQoL was assessed with the Pediatric Quality of Life Inventory version 4.0 Generic Core Scales (PedsQL™4.0) (Varni et al., 2001). Items are divided into 4 domains: physical health, emotional functioning, social functioning and school functioning. Two summary scores are computed: psychosocial health (emotional, social and school functioning) and total score (all domains). Scores range from 0 to 100, with higher scores indicating better HRQoL. Internal consistency for the PedsQL was satisfactory across time points, with Cronbach's α = 0.69 to α = 0.95.

*Socio-demographic characteristics* were obtained from parents via an online socio-demographic questionnaire at baseline: family composition, socioeconomic status (income), gender and ethnicity. Adolescent's stressful life-events and the use of psychological care besides *Op Koers Online* were also obtained.

Within the same questionnaire, *illness characteristics* (illness type, duration, severity) were obtained. Parents rated illness severity using a proxy measure based on the occurrence of the following 13 possible consequences of CI in the past year (scale 0–13): doctor visits, hospitalization, surgery, use of medication, dietary consequences, visible malformations, non-visible malformations, use of appliances, limitations in movements, problems with hearing, vision and speech (0 = no, 1 = yes) and course of the disease (0 = improving/stable, 1 = deterioration/unstable).

### 2.3 Statistical analysis

A priori power analysis based on the detection of an intervention effect of medium size with d *≈* *0*.50, indicated a required number of 84 study participants. Post-hoc power calculations based on the inclusion rates of the current study with three time points indicated that differences of medium size (*d* = 0.65) between study groups over time at a significance level of 0.05 with a power of 0.80 could be detected (Twisk, 2013). Preliminary analyses examined baseline differences between the intervention and control group on socio-demographic and illness characteristics and on the outcome variables. To characterize the sample, externalizing problems and HRQoL at T0 were compared to Dutch norms (Verhulst et al., 1996; Verhulst et al., 1997; van Muilenkom et al., 2021) with one-sample tests. Comparison of internalizing problems was not possible because norm scores were not available for internalizing problems without somatic complaints.

Linear mixed model analyses were performed to examine efficacy of the intervention accounting for dependency of data within participants. Correcting the analyses for dependency within study groups was not necessary, as the intra-class correlation coefficients were not significant (or below 0.05) (Blozis, 2003). Outliers on outcome measures were rescaled according to Tabachnick and Fidell (2013). Intention-to-treat analyses were performed based on the random allocation, using the mixed-model procedure in SPSS (19.0) with Full Maximum Likelihood estimation. Participants were included in the efficacy analyses if data at baseline (T0) were available as well as data at T1 and/or T2. Missing data were not imputed. To facilitate interpretation of regression coefficients, all continuous scores were standardized, expressing deviations from the mean at T0. For binary coded variables, standardized regression coefficients of 0.2 were considered small, 0.5 medium and 0.8 large (Cohen, 1998).

Dependent variables were parent- and self-reported internalizing and externalizing problems disease-related coping skills and HRQoL. Linear mixed models were fitted with a random intercept and fixed slopes for study group (intervention vs control), time (T1 vs T0 and T2 vs T0) and the interaction term study group × time. This interaction tested the effects of the intervention. Potential differences between intervention and control group on outcome measures at T0 were controlled by the random intercept. An alpha of 0.05 was used to test the statistical significance of the effects.

## 3 Results

### 3.1 Sample characteristics

**Fig. 2:**
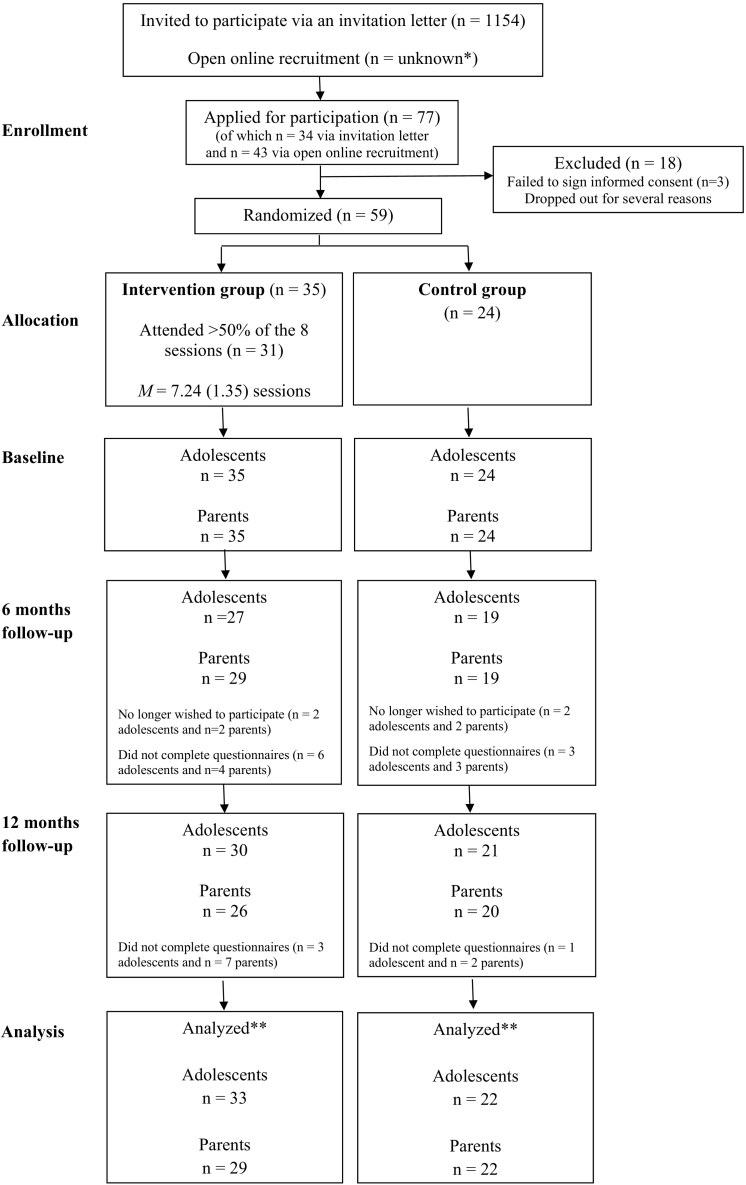
Participant flow through the study. * Open recruitment was done via patient associations, social media and advertisements on websites. The number of adolescents reached online is unknown. ** Linear mixed model analyses included all available data from each subject with a baseline and at least one follow-up assessment up to withdrawal or study completion. Note: Parents were not involved in the intervention. They were asked to fill out a socio-demographic questionnaire once and a questionnaire about their child at each time-point.

Fig. 2 shows the participant flow from recruitment to follow-up. Most applicants (56%) applied after seeing online advertisement. Of those who received an invitation letter, 3% applied. Of the applicants, 23% dropped out before randomization. Main reason for drop-out was that they were not available at the times scheduled for the sessions.

Regarding the intervention, course leaders reported no study-related adverse events. As recorded by the course leaders in the log, 88% of all sessions was carried out according to the protocol. The sessions that were not, concern the first session in which a lot of time was spent on explaining the intervention and getting to know each other. Course leaders were advised to do so for creating group cohesion and, when necessary, move some treatment elements to the next session.

In total, 59 participants were randomized, of which 35 were assigned to the intervention group and 24 to the control group. A total of 31 (89%) participants from the intervention group attended more than 50% of the sessions. Mean attendance was 7.2 (SD = 1.4) sessions. In the end, 55 participants were included in the analyses, of which 33 in the intervention group (Mean age = 15.1 SD = 1.7; 45.5% female) and 22 controls (Mean age = 15.0 SD = 1.8; 63.6% female). Compared to the Dutch norms (*p* < .05), our sample reported significantly fewer problems on baseline self-reported externalizing problems (Verhulst et al., 1997) and worse HRQoL (physical, psychosocial, emotional, social, school and total) (van Muilenkom et al., 2021). At baseline, 13% of participants scored in the clinical range for internalizing problems; 5% of the participants scored in the clinical range for externalizing problems. There were no significant differences in socio-demographic characteristics at baseline between the intervention and control group (Table 2). The intervention group had significantly worse HRQoL (physical, social, school, psychosocial and total) than the control group at baseline.

**Table 2:** Sample socio-demographic characteristics.

	Intervention group (*N* = 33)	Control group (*N* = 22)	*p*^a^
Age *(in years)*	15.1 (1.7)	15.0 (1.8)	0.824
Gender			0.186
Male	54.5%	36.4%	
Female	45.5%	63.6%	
Income			0.390
< Modal	24.1%	14.3%	
≥ Modal	75.9%	85.7%	
Ethnicity (based on country of birth of the parent of the participant)			0.230
Dutch	72.7%	86.4%	
Non-Dutch	27.3%	13.6%	
Diagnosis			
Asthma	18%	23%	
Type 1 diabetes	18%	14%	
IBD	3%	9%	
Auto-immune disease (JIA)	6%	5%	
Hashimoto's disease	3%	9%	
Other^b^	52%	40%	
Illness duration (*in years)*	8.66 (5.79)	7.95 (5.22)	0.651
Illness severity *(scale 0–13)*	4.06 (1.72)	3.55 (1.14)	0.658
Former psychological help			0.849
Yes	20%	17.6%	
No	80%	82.4%	

a Group differences tested with independent samples *t*-tests for continuous variables and χ2-tests for categorical variables.

b All other diagnoses occurred once.

During the intervention period, 11% (*n* = 2) of the adolescents in the control group received alternative psychosocial care (individual psychological treatment). At 6-months follow-up, 11% in the control group and 14% in the intervention group received additional psychosocial care. At 12-months follow-up, this was 14% in the control group and 28% in the intervention group; these percentages did not differ significantly (*p* = .26).

### 3.2 Primary outcomes

#### 3.2.1 Internalizing and externalizing problems (CBCL, YSR)

The intervention had no significant effect on the change in parent-reported and self-reported internalizing or externalizing problems over time (Table 3). None of the interactions of study group × time were significant.

**Table 3:** Means, Standard Deviations at baseline (T0), 6-month (T1) and 12-month follow-up (T2) and results of linear mixed model analyses.

	Intervention group			Control group			Study group*time			
	T0	T1	T2	T0	T1	T2	T0-T1 intervention vs waitlist		T0-T2 intervention vs waitlist	
	M (SD)	M (SD)	M (SD)	M (SD)	M (SD)	M (SD)	β	*p*	β	*p*
Behavioral problems (YSR)	N = 33	*N* = 27	*N* = 30	N = 22	*N* = 19	*N* = 21				
Internalizing (without somatic complaints)	9.39 (5.29)	9.78 (7.72)	9.90 (6.82)	7.50 (6.14)	8.00 (6.46)	8.24 (7.01)	−0.34	0.140	−0.24	0.287
Externalizing	8.67 (6.37)	7.15 (6.04)	7.07 (4.47)	6.27 (4.14)	7.21 (5.82)	6.62 (4.85)	−0.34	0.129	−0.28	0.195

Behavioral problems (CBCL)	*N* = 29	N = 29	*N* = 26	*N* = 23	N = 19	*N* = 20				
Internalizing (without somatic complaints)	7.48 (7.25)	5.93 (5.41)	6.65 (6.24)	7.74 (5.31)	6.53 (5.56)	5.45 (5.40)	−0.02	0.928	0.21	0.354
Externalizing	5.17 (5.54)	4.14 (4.16)	5.08 (4.70)	5.96 (3.82)	5.89 (5.95)	4.30 (3.61)	−0.05	0.815	0.32	0.140

HRQoL (PedsQL)	N = 33	N = 27	N = 30	N = 22	N = 19	N = 21				
Physical health	59.85 (22.25)	68.40 (22.22)	65.94 (24.28)	78.27 (18.84)	77.63 (20.92)	80.21 (18.26)	0.34	0.056	0.17	0.309
Emotional functioning	69.09 (18.48)	71.67 (18.55)	71.17 (18.55)	75.91 (19.00)	70.53 (20.06)	70.95 (23.80)	0.35	0.212	0.29	0.272
Social functioning	71.82 (17.63)	76.67 (15.81)	76.17 (17.75)	87.95 (13.42)	82.89 (15.75)	85.00 (16.12)	**0.56**	0.011	0.41	0.052
School functioning	60.61 (17.22)	63.70 (17.90)	60.50 (17.14)	72.27 (15.41)	65.26 (16.20)	66.90 (17.50)	**0.55**	0.029	0.31	0.198
Psychosocial functioning	67.17 (13.66)	70.68 (14.53)	69.28 (14.04)	78.71 (12.92)	72.89 (15.12)	74.29 (16.13)	**0.60**	0.017	0.42	0.081
Total HRQoL	64.62 (15.09)	69.89 (15.55)	68.12 (16.53)	78.56 (13.66)	74.54 (16.09)	76.35 (16.06)	**0.52**	0.015	0.33	0.107

Disease-related coping skills (OKQ)	N = 33	N = 27	N = 30	N = 22	N = 19	N = 21				
Information seeking	1.97 (0.60)	2.51 (0.45)	2.45 (0.61)	2.23 (0.61)	2.41 (0.63)	2.34 (0.62)	0.52	0.063	**0.61**	0.026
Use of relaxation	1.77 (0.71)	2.21 (0.66)	2.06 (0.71)	1.89 (0.70)	1.84 (0.83)	1.95 (0.70)	**0.68**	0.011	0.30	0.232
Social competence	1.72 (0.62)	2.12 (0.44)	1.94 (0.59)	1.79 (0.61)	1.80 (0.70)	1.92 (0.64)	**0.57**	0.030	0.13	0.613
Positive thinking	1.73 (0.74)	2.15 (0.68)	2.20 (0.61)	1.70 (0.86)	1.95 (0.75)	1.92 (0.87)	0.21	0.521	0.31	0.325

Note: Significant (*p* < .05) intervention effects (β) are presented in bold.

#### 3.2.2 Disease-related coping (OKQ)

**Fig. 3:**
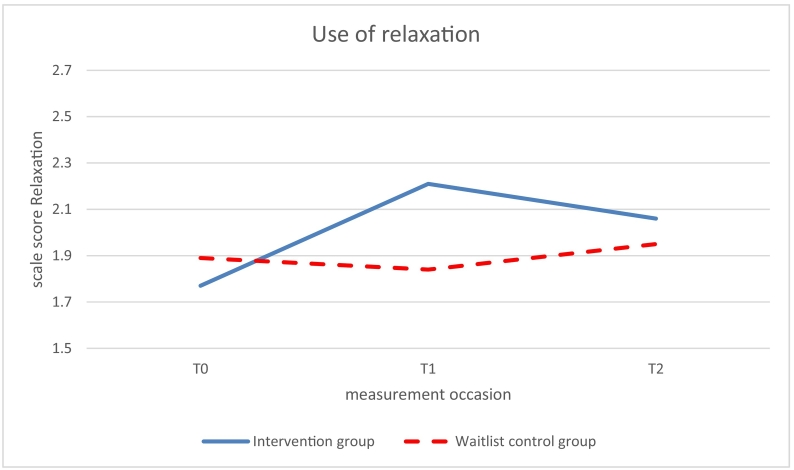
Use of the disease-related coping skill relaxation measured with the Op Koers Questionnaire, at T0 (baseline), T1 (6 months follow-up), and T2 (12 months follow-up); intervention (Op Koers Online) and waitlist control group.

**Fig. 4:**
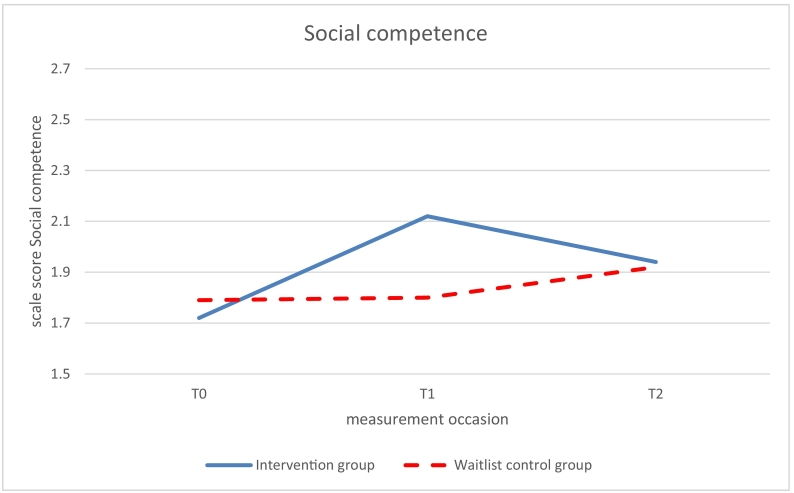
Use of the disease-related coping skill social competence measured with the Op Koers Questionnaire, at T0 (baseline), T1 (6 months follow-up), and T2 (12 months follow-up); intervention (Op Koers Online) and waitlist control group.

**Fig. 5:**
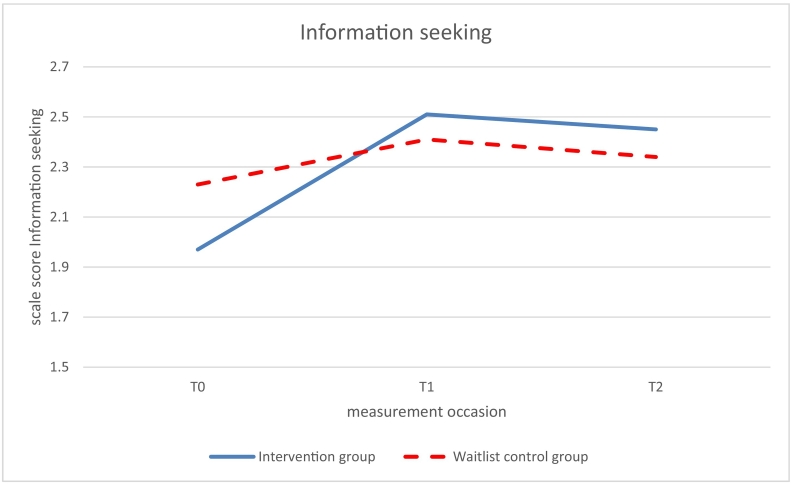
Use of the disease-related coping skill information seeking measured with the Op Koers Questionnaire, at T0 (baseline), T1 (6 months follow-up), and T2 (12 months follow-up); intervention (Op Koers Online) and waitlist control group.

Significant beneficial effects of the intervention (study group x time *p* < .05) on use of coping skills were found at 6-months follow up (T1 vs T0) for relaxation (β 0.68, *p* = .0.011) and social competence (β = 0.57, *p* = .030; see Table 3, Fig. 3, Fig. 4). The intervention group improved over time while the control group did not improve. No significant effects on relaxation and social competence were found at 12-months follow-up (T2 vs T0). The intervention effect on information seeking was marginally significant at 6-months follow up (β = 0.52, *p* = .063) but significant at 12-months follow up (β = 0.61, *p* = .026; Fig. 5).

### 3.3 Secondary outcomes

#### 3.3.1 Health-related quality of life (PedsQL)

**Fig. 6:**
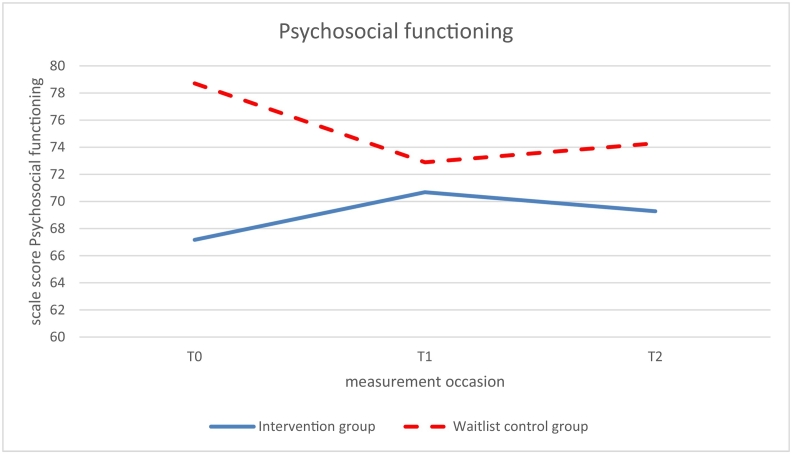
Psychosocial functioning measured with the Pediatric Quality of Life Inventory version 4.0 Generic Core Scales (PedsQL™4.0), at T0 (baseline), T1 (6 months follow-up), and T2 (12 months follow-up); intervention (Op Koers Online) and waitlist control group.

**Fig. 7:**
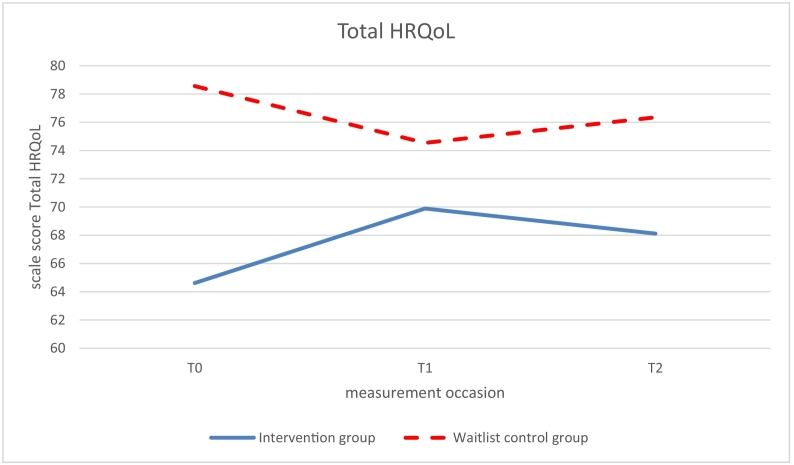
Total Health-Related Quality of Life measured with the Pediatric Quality of Life Inventory version 4.0 Generic Core Scales (PedsQL™4.0), at T0 (baseline), T1 (6 months follow-up), and T2 (12 months follow-up); intervention (Op Koers Online) and waitlist control group.

Significant beneficial effects of the intervention (study group × time, *p* < .05) were found at 6-months follow up (T1 vs T0) on social functioning, school functioning and on the summary scales psychosocial health (Fig. 6) and total HRQoL (Fig. 7); β ranging from 0.52–0.60, *p* ranging from 0.011 to 0.029 (Table 3). While HRQoL in the intervention group improved from T0 to T1, HRQoL of the control group did not improve from T0 to T1 or worsened.

No significant effects were found at 12-months follow-up (T2 vs T0), though the marginal significance of the regression coefficients of social functioning (β = 0.41, *p* = .052) and psychosocial functioning (β = 0.42, *p* = .081; Fig. 5) indicated a long-term effect of the intervention at T2.

## 4 Discussion and conclusion

### 4.1 Discussion

The results of this RCT indicate a positive effect of *Op Koers Online* for adolescents, an online psychosocial group intervention for adolescents with different types of CI: the intervention had a positive effect on disease-related coping skills (use of relaxation, social competence and information seeking) and on HRQoL (social, school, psychosocial and total). The effect sizes were medium with standardized regression coefficient β's between 0.52 and 0.68, which was in line with earlier studies on the efficacy of psychosocial interventions for youth with a CI (Scholten et al., 2013; Barakat et al., 2010).

We found no statistically significant intervention effect on parent- and self-reported internalizing and externalizing problems in the present RCT nor in the previous pilot study on *Op Koers Online for adolescents*. Possibly, this is due to the fact that *Op Koers Online* is a preventive as well as curative intervention (Douma et al., 2019) so that *Op Koers Online* is not explicitly designed to decrease psychopathology symptoms. Furthermore, having clinical levels of behavioral problems was not an inclusion criterion, thereby limiting the chance to achieve a decrease in internalizing and externalizing problems by the intervention. The absence of intervention effects on internalizing and externalizing problems in the present study is not in line with the results of the previous RCT on *Op Koers face-to-face*. Since the intervention effects of *Op Koers face-to-face* were of small size (Last et al., 2007), just as the effects in the present RCT on *Op Koers Online*, one could assume that lack of power in the present study explained the discrepancies between the statistically significant effects of *Op Koers Online* and *Op Koers face-to-face*.

Recruitment was problematic. The response rate on information letters was lower than expected based on former research on *Op Koers* face-to-face (Scholten et al., 2013). Even though we experienced that face-to-face recruitment works best, healthcare professionals had trouble motivating adolescents to participate in the study. Therefore, the intended sample size was not reached. Inclusion problems in RCT's, especially with adolescents, are common (Watson and Torgerson, 2006). Given their age and puberty, adolescents are a hard group to motivate for any intervention, possibly especially a psychosocial group intervention. However afterwards, we found that the adolescents enjoyed the intervention. Satisfaction was high and two-third of the participants would definitely recommend the intervention to peers, while a quarter reported that they maybe would recommend the intervention (data not shown). Healthcare providers should be (more) aware of this, and should pay more attention explaining to adolescents what the intervention entails and why participation could be helpful. Next to medical doctors, it is important to involve nurses in this process. Furthermore, it is important to note that engaging participants in the design and delivery of interventions and integrating their feedback may increase participants' engagement in interventions.

The intervention effect on coping skills diminished at one-year follow-up with exception of information seeking and giving about the disease, which is an important factor for good adaption to living with the disease. In the study of Scholten et al. (2013) regarding *Op Koers face-to-face*, the long-term intervention effects were stronger when parents were involved. In the face-to-face intervention, parents and children/adolescents participated in separate, parallel groups and parents learned how to support their child in daily life. *Op Koers Online* for adolescents was intentionally designed without involving parents, with the argument that it keeps the threshold for participation low and gives adolescents the opportunity to participate independently from their parents. However, separately from the intervention for adolescents, the *Op Koers Online* program offers a module for parents, *Op Koers online for parents*. It aims to prevent and/or reduce psychosocial problems of parents by teaching the adaptive coping skills related to their child's disease. *Op Koers online for parents* was recently positively evaluated (Douma et al., 2020). *Op Koers Online for parents* intervention can be recommended to parents of participating adolescents, to support parents in how to cope with their child's CI. Future research should focus strengthening the long-term effects by involving parents.

With this RCT, we contribute to the literature with an evaluation of a unique online CBT group intervention for adolescents with different types of CI. The study has some limitations. First limitations are the unknown recruitment and enrollment rates and the lack of information about non-respondents, cause of partially online open recruitment. Second, we relied on self-reported outcome measurements, which has the risk for socially desirable answers or concealing of symptoms. This could have led to an overestimation of the intervention effect and should be taken into account while interpreting the results. Furthermore, the outcomes relaxation and positive thinking had moderate internal consistency. On the one hand, the use of scales with moderate internal consistency is acceptable for group comparisons because the internal consistency is an indication of random error and has nothing to do with systematic error (bias). On the other hand, Cronbach's alphas should preferably be 0.7 or higher because the lower the internal consistency, the larger the random measurement error, and so, the more difficult to detect differences between groups.

A priori power analysis was based on the detection of an intervention effect of medium size with *d* ≈ 0.50, while the post hoc power analysis revealed that with the current sample size we were still able to detect an intervention effect of medium size with *d* = 0.65. Furthermore, there were differences between participants in the two study groups on HRQoL at baseline. The mixed models analyses corrected for baseline differences between intervention and controls but the intervention group might have had more room for improvement.

Future efforts should focus on maintaining the effects on coping skills at one-year follow-up and it would also be interesting to investigate whether the effects of the intervention on HRQoL and are mediated by the disease-related coping skills taught during the intervention.

### 4.2 Conclusion

The results of this randomized controlled trial indicate a positive effect of an innovative online psychosocial group intervention for adolescents with all kinds of CI. After following the intervention, participant's use of adaptive coping skills and their HRQoL improved.

### 4.3 Practice implications

The *Op Koers Online* intervention is an important addition to the pediatric field to support adolescents with a CI, and contribute to their HRQoL. A big advantage is the possibility to participate from home, so that additional hospital visits are not necessary. Healthcare professionals (medical doctors, nurses, etc.) should be involved in recruitment, and should be aware of the necessity of motivating adolescents to participate. The *Op Koers Online for parents* intervention can be offered to parents of participating adolescents (Douma et al., 2020). The next step is to support more adolescents by using *Op Koers Online* for adolescents in regular clinical practice. At the same time, the intervention needs to be investigated more widely to keep improving the content and confirm the positive outcomes.

## Abbreviations

CI: chronic illness
RCT: randomized controlled trial
HRQoL: health related quality of life

## Funding source

This study was funded by 10.13039/501100003142Fonds NutsOhra (FNO; project number: 100.977), a social fund for vulnerable groups in Dutch society. FNO had no role in the study design.

## Clinical trial registry name

ISRCTN.

## Registration number

ISRCTN83623452.

## Declaration of competing interest

The authors declare that they have no known competing financial interests or personal relationships that could have appeared to influence the work reported in this paper.
